# Fractal Analysis as a Predictor of Early Implant Loss: A Retrospective Study

**DOI:** 10.1016/j.identj.2025.103880

**Published:** 2025-08-30

**Authors:** Dóra Iványi, Márton Kivovics, Richárd Balázs, Orsolya Németh

**Affiliations:** Department of Public Dental Health, Semmelweis University, Budapest, Hungary

**Keywords:** Early implant failure, Fractal analysis, Fractal dimension, lacunarity, Cone beam computed tomography, Panoramic radiography

## Abstract

**Introduction and aims:**

Fractal dimension (FD) and lacunarity are increasingly applied to assess trabecular bone structure, yet their predictive value for early dental implant failure remains uncertain. This study investigated whether FD and lacunarity, measured on panoramic radiographs and cone-beam computed tomography (CBCT), can predict early implant loss in the mandible.

**Methods:**

A retrospective case-control study included 48 patients—24 with early implant failure and 24 with implants surviving at least 5 years without periimplantitis. All had panoramic radiographs, and a subset of 30 patients (15 per group) also had CBCT scans. FD and lacunarity were assessed in 3 mandibular regions: frontal, premolar, and molar. ImageJ software with box-counting method was used. FD from panoramic images was calculated using the White and Rudolph method; CBCT-based FD values were derived using both the White and Rudolph and the Kato et al. methods.

**Results:**

No significant differences in FD or lacunarity values were found between groups in any region on either imaging modality (*P > .*05).

**Conclusions:**

Fractal dimension and lacunarity values obtained from panoramic radiographs and CBCT images did not demonstrate predictive capability for early implant failure. Although fractal analysis offers a non-invasive approach to assess trabecular bone architecture, it should not be used in isolation to estimate implant prognosis.

**Clinical Relevance:**

While fractal analysis may provide complementary insights into bone microstructure, its standalone application lacks sufficient reliability for predicting early dental implant failure. Future studies involving larger cohorts are warranted.

## Introduction

Dental implants are widely used for the rehabilitation of partial and complete edentulism, with high reported survival rates exceeding 90% over 10 years.^1^, ^2^, ^3^ Despite overall clinical success, early implant failure remains a relevant complication, with incidence rates ranging from 1% to 5% depending on patient- and site-specific factors.^4^, ^5^, ^6^ Early implant failure is typically defined as the loss or removal of an implant prior to functional loading. Unlike late failures, which are often associated with peri-implantitis, mechanical overload, or prosthetic complications, early implant failure is usually attributed to impaired osseointegration, where the direct bone-to-implant contact either partially or completely fails to establish during the initial healing phase. In the event of early implant failure, instead of osseointegration, a fibro-osseous connection is established between the implant and the bone.^7^^,^^8^ Diagnosis of early implant failure is based on clinical signs such as implant mobility, persistent pain, infection, or the absence of radiographic evidence of bone integration.^4^

The aetiology of early implant failure is multifactorial. Key contributing factors include surgical trauma, bacterial contamination, poor vascularization, systemic conditions (e.g., uncontrolled diabetes mellitus, smoking), local anatomical characteristics and the surface properties of the implant, such as roughness, wettability, and chemical composition.^4^^,^^9^, ^10^, ^11^, ^12^ Among these, bone quality plays a particularly crucial role. Adequate bone density and trabecular architecture are essential for achieving primary implant stability, which is widely recognized as a prerequisite for successful osseointegration.^13^^,^^14^ However, bone quality assessment in clinical practice is often subjective—typically relying on tactile feedback during drilling—thus highlighting the need for objective, reproducible, image-based methods to evaluate the microstructural properties of bone.

Fractal analysis has emerged as a promising method for the quantitative evaluation of bone microarchitecture. The fractal dimension (FD) quantifies the complexity and self-similarity of trabecular patterns, providing insight into bone heterogeneity beyond simple density measures.^15^ Higher FD values generally correspond to more complex and better-connected trabecular networks, which are thought to be favourable for implant stability.^16^ Lacunarity, a complementary parameter, measures the texture and spatial distribution of gaps or voids within the trabecular structure, offering additional information about bone uniformity and porosity.^17^^,^^18^ While FD reflects structural complexity, lacunarity reflects the “gappiness” or heterogeneity of the structure—thus, both parameters together may provide a more complete understanding of bone quality.^19^

Traditionally, fractal analysis has been applied to 2-dimensional radiographic images, such as periapical or panoramic radiographs, due to their accessibility and low radiation dose.^20^, ^21^, ^22^ Recently, there has been growing interest in extending FD analysis to 3-dimensional imaging modalities, especially cone-beam computed tomography (CBCT).^23^ Despite the higher spatial resolution and volumetric data provided by CBCT, standardization and validation of FD and lacunarity analysis in 3D datasets remain limited, partly due to voxel size variation, noise, and segmentation challenges.

Several studies have investigated the potential association between trabecular bone microarchitecture (quantified by FD) and early dental implant failure.^24^, ^25^, ^26^ The hypothesis behind this line of research is that bone with lower FD values, indicative of reduced structural complexity and connectivity, may be less capable of achieving or maintaining osseointegration during the early healing phase.^27^ Implant stability and radiodensity assessments, such as Implant Stability Quotient (ISQ) and Hounsfield unit (HU) values, have also been associated with implant outcomes, and may provide additional insight when combined with image-based bone quality metrics Hounsfield unit.^28^, ^29^, ^30^ However, such clinical parameters are often unavailable in retrospective designs, limiting their integration in studies like ours.

Although CBCT imaging provides volumetric data, most studies utilizing fractal analysis on CBCT datasets continue to rely on 2-dimensional slice-based evaluations rather than true 3-dimensional volumetric analysis. Only a few experimental studies have performed full 3D fractal assessments, emphasizing the need for further development and standardization in this field.^21^^,^^26^, ^27^, ^28^ While Hounsfield unit–based bone density estimation has been used in preoperative planning, recent trends emphasize structural assessment over density, with methods such as fractal analysis gaining importance.^28^ The novelty of this study lies in the volumetric 3D analysis of general mandibular trabecular bone, rather than peri-implant areas, and in combining FD and lacunarity assessment across multiple anatomical regions. To our knowledge, this systemic, retrospective application of CBCT-based 3D fractal analysis remains rare in early implant failure research.

The aim of our retrospective case-control study was to examine whether fractal dimension and lacunarity analysis of panoramic radiographs and CBCT reconstructions can be utilized for predicting early implant failure.

Our null hypothesis states that there is no significant difference between the fractal dimension and lacunarity values measured on panoramic radiographs and CBCT reconstructions of patients with early implant loss compared to those with surviving implants.

## Methods

### Participants

This study was approved by the Semmelweis University's Regional Research Ethics Committee (189/2024) and was conducted according to the Declaration of Helsinki. The reporting of this study confirms to the Strengthening the Reporting of Observational studies in Epidemiology (STROBE) guidelines. The checklist for this study is presented in Supplementary Table 1. The present case-control retrospective study included patients treated at the Department of Public Dental Health, Semmelweis University between 2015 and 2025. Data collection and analysis was performed in April 2025. Patients were informed about the surgical and prosthetic interventions and signed informed consent forms.

*Inclusion criteria for patients undergoing dental implant placement:*

• Patients over 18 years old
• Requiring dental implants for their oral rehabilitation were considered as candidates for implant placement

*The exclusion criteria for patients undergoing dental implant placement:*

• Uncontrolled medical conditions that may alter bone metabolism (osteoporosis, Cushing's syndrome, hypophosphatasia, osteogenesis imperfecta, osteomalacia, Paget's disease, osteopenia, osteofibrosis, hyperparathyroidism, hypophosphatemia, Vitamin D deficiency, skeletal dysplasias, chronic renal failure, uncontrolled diabetes mellitus)
• Receiving medication that could affect bone metabolism (Antiresorptive agents (bisphosphonates, denosumab, monoclonal antibodies, VEGF inhibitors), glucocorticoids, calcitonin or parathyroid hormone, Herapin therapy for months, cyclosporine, high-dose medroxyprogesterone acetate therapy, chemotherapeutic agents (methotrexate, ifoxamide, imatinib), thiazolidinediones, antiretroviral therapy (anti-HIV drugs)
• Chemotherapy
• Therapeutic doses of irradiation treatment directed at the head and neck region
• Localized periapical disease
• Evidence of uncontrolled periodontal disease
• Recreational drug abuse
• Anticoagulant treatment that contraindicates implantation or surgical interventions
• History of psychological instability,
• Physical or intellectual disability,
• Alcoholism or heavy smoking.
• Pregnancy or nursing

*Inclusion criteria for patients the test group:*

The test group included patients who received implants in the Department and exhibited early implant failure.

• Implant failure was considered as early implant failure if no intimate bony connection was formed between the implant surface and the recipient site and implants were lost prior to the connection of the supraconstruction^31^, ^32^, ^33^

*Exclusion criteria for patients the test group:*

• Patients whose implants had been temporarily or permanently restored or loaded
• Patients whose implants had to be removed after implant placement due to malposition, damage of surrounding anatomical landmarks or biomechanical complications

Patients in the control group were randomly included from a pool of patients with surviving implants placed at the Department to match the age and sex distribution of the patients included in the test group.

*Inclusion criteria for patients in the control group:*

• Patients with surviving implants for at least 5 years
• No signs of progressive peri-implantitis. The diagnosis of peri-implantitis is based on the guidelines of Renvert et al.^34^ No presence of inflammation has been registered in these patients after clinical and radiological examinations during follow-up examinations.

### Surgical interventions

Dental inflammations (e.g. periodontal, periapical inflammations) were treated before radiographs were taken, and local contraindications of implant placement were eliminated. All patients received professional oral prophylaxis prior to treatment. Dental implant placement was performed at least 2 months after the tooth extractions at the implant recipient sites. Dental implant placement was performed under local anesthesia according to the instructions of the manufacturer of the implant. In this study 2 implant types were placed Callus Pro (Callus Implant Solutions, Nürnberg, Germany) and Nobel Replace Parallel Conical Connection (Nobel Biocare, Kloten, Switzerland). Patients were properly informed about post-operative care and were prescribed antibiotic therapy (2 × 1 amoxicillin/clavulanic acid (875 mg/125 mg) for 7 days, 4 × 1 clindamycin (300 mg) for 7 days in case of penicillin allergy.) Sutures were removed after 1 week. A conventional loading approach was applied; patients did not wear temporary prostheses, and final prostheses were fabricated after a 2-month-long healing period.

Dental and general patient history, preoperative panoramic radiographs and CBCTs were collected and analyzed.

### Radiographic image analysis

The panoramic X-rays were taken with KAVO OP3D PRO (KAVO, Biberach an der Riß, Germany) hardware with a tube voltage of 66.42 kV, a tube current of 10 mA, an exposition of 16.18 s, and a focus detector distance of 50 cm using the same protocol. Pre-implantation CBCT’s were taken from the mandible with the PaX-Reve3D (Vatech, Hwaseong, South Korea) of patients was performed with an isotropic voxel size of 200 µm, 360 ° rotation, 94 kV tube voltage, 7.2 mA tube current, 9 s exposition duration, and a 15 × 15 cm field of view to obtain Digital Imaging and Communications in Medicine (DICOM) data. While orthopantomograms were available for all 24 patients in the test group, CBCTs from the mandible were limited to 15 of these cases. Accordingly, in the control group, all 24 patients had their measurements obtained from panoramic radiographs, but only 15 of these patients had their data calculated from CBCTs.

Prior to fractal analysis, orthopantomograms and CBCTs must also undergo prior image manipulation. Image analysis was performed using the ImageJ software version 1.54p bundled with 64-bit Java1.8.0_322 (Rasband, W., National Institutes of Health, Bethesda, Maryland, USA, https://imagej.nih.gov/ij).^35^

### 2D image analysis

**Fig. 1:**
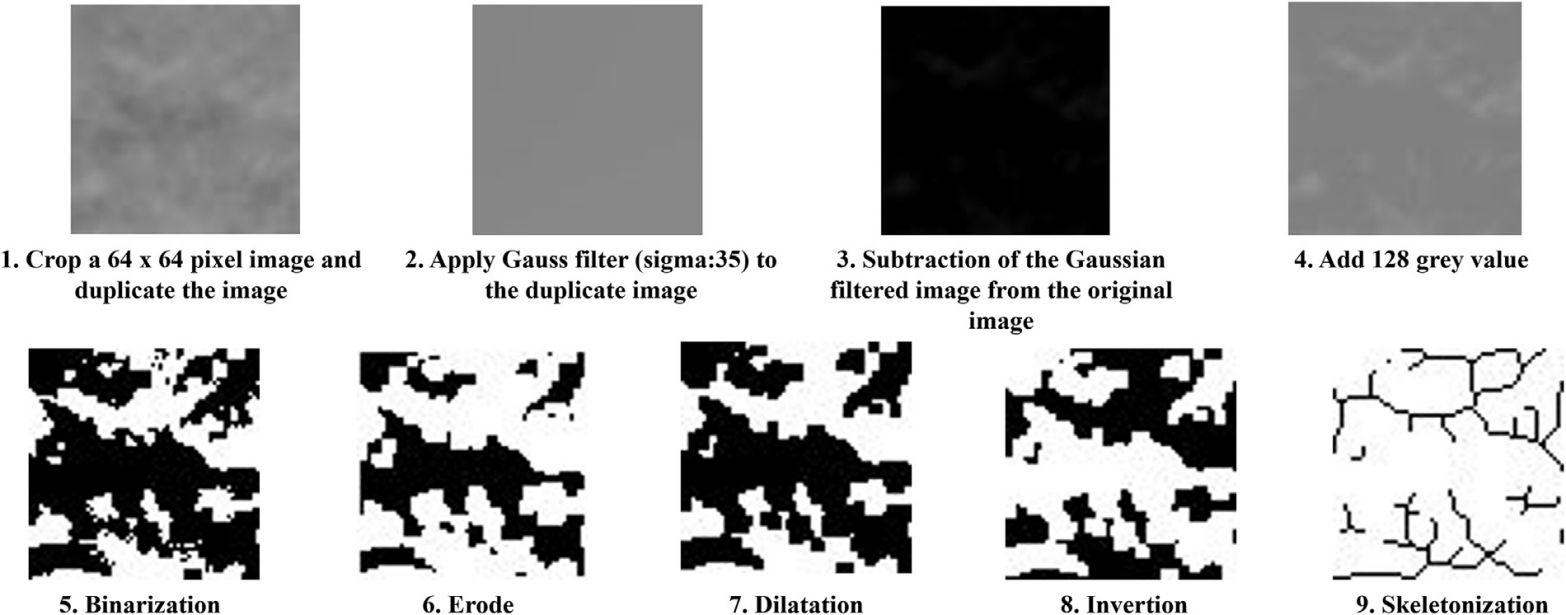
Steps of 2D image manipulation. A sequence illustrating the preprocessing steps for fractal analysis on panoramic radiographs. The process includes cropping a 64 × 64 pixel region of interest (ROI), Gaussian blurring, subtraction, grayscale adjustment, binarization, erosion, dilation, inversion, and final skeletonization using ImageJ and the FracLac plugin.

Fractal analysis was performed and lacunarity was measured on the panoramic radiographs of 24 patients per group (48 patients altogether) in 3 locations (front, premolar, molar). After saving the panoramic radiographs in TIFF format, 3 64 × 64 pixel region of interest (ROI) was cropped on the mandible. ROIs were selected in the frontal, premolar and molar regions. ROIs were not allowed to include anatomical features, mandibular basis, inflammation, tooth, implant and other bone lesions. Measurements were generally performed on the right side of the jaw, but if a lesion prevented a proper ROI, image analysis was performed on the left side. Image analysis was carried out using the method of White and Rudolph.^20^ The cropped ROI was duplicated, and a Gaussian blur (σ = 35) was applied to the duplicated ROI to reduce the X-ray brightness due to soft tissue and bone thickness. The filtered ROI was subtracted from the original ROI and 128 grey values were added. The image was then binarized with a threshold value of 128, resulting in values less than 128 gray values being converted to black, while those with higher values were converted to white on the image. The image was then eroded and dilated to reduce radiographic noise. Then, it was inverted and finally skeletonized, where the black contours were based on the trabecular bone pattern. Skeletonization reduces the trabecular image to a 1-pixel-wide representation of the bone structure, allowing topological analysis of connectivity and complexity. This representation forms the basis for calculating the fractal dimension using box-counting methods. From the skeletonized image, fractal analysis was performed via the FracLac plugin^36^ version 2.5 (Karperien, A., Charles Strut University, Australia) using the box counting method (box sizes: 2, 3, 4, 6, 8, 12, 16, 32, 64) and the fractal dimension value of the image was obtained.^20^ In addition to FD, the lacunarity value was also calculated^18^ (Figure 1).

### 3D image analysis

**Fig. 2:**
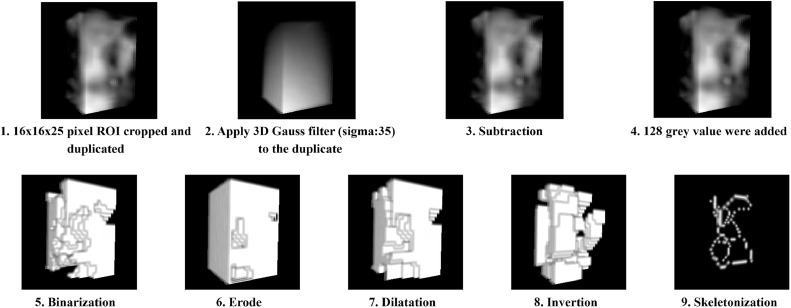
Steps of 3D image manipulation using the White and Rudolph method. Representative example of image preprocessing applied to CBCT volumes using the White and Rudolph protocol. The process involves cropping a 16 × 16 × 25 voxel ROI, 3D Gaussian blur, subtraction, grayscale adjustment, binarization, erosion, dilation, inversion, and 3D skeletonization prior to fractal analysis using the BoneJ plugin.

Fractal analysis was performed on the CBCT reconstructions of 15 patients per study group (altogether 30 patients) using 2 different methods. The 3D image analysis was also performed in ImageJ. The CBCT images in DICOM format were imported as image sequences and saved in TIFF format. Measurements on the CBCTs were performed from similar locations as for the panoramic radiographs; the frontal, premolar and molar regions. The ROI was defined in 2 steps; first, a 16 × 16 pixel area was selected in the transversal plane and then cropped. Then, using the "reslice" function, the images were converted to the sagittal plane, and again a 16 × 25 pixel region was selected and cropped. The reason for the smaller ROI size selection is that the mandibular thickness varies greatly, but in each patient, we found 16 × 16 × 25 pixels of pure trabecular bone tissue in the study areas. Image manipulation was performed using 2 different methods. First, we used the technique described by White and Rudolph.^20^ Using BoneJ plugin,^37^ a 3D Gaussian blur was added to the ROI (σ = 35) and the blurred images were subtracted from the original ROI. A grey value of 128 was added to the resulting images and then binarized, eroded, dilated, inverted and 3D skeletonization was applied using BoneJ. Finally, the fractal dimension value was obtained using the BoneJ plugin (Figure 2).

**Fig. 3:**
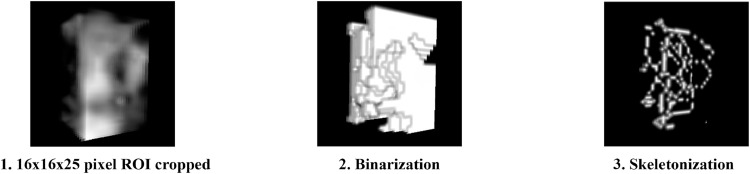
Steps of 3D image analysis based on the Kato et al. method. Simplified workflow for fractal analysis of CBCT data using direct binarization and 3D skeletonization. This method omits Gaussian filtering and morphological processing, assuming minimal distortion in CBCT datasets.

While several steps of image manipulation as described by White and Rudolph^20^ are necessary to minimize the overlapping X-ray shadows and X-ray distortion, it is assumed that these steps are not required for CBCT scans, since these distorting effects are not present. For these reasons, we also performed the simplified image manipulation described by Kato et al.^38^ The cropped ROI was binarized with an optimized threshold, then skeletonized using the BoneJ plugin and fractal analysis was performed (Figure 3).

### Statistical analysis

Data analysis was performed using IBM SPSS Statistic software, version 29 (IBM Corporation, New York, NY, USA). The normal distribution of the analysed data was determined using the Kolmogorov-Smirnov and Shaphiro-Wilk tests. If the data showed normal distribution, an independent samples t-test was performed, while in the absence of normal distribution, Mann-Whitney U test was used to examine the variations. The level of significance was set at 0.05.

All individual-level and summary data used in the analysis are openly available at Mendeley Data at https://doi.org/10.17632/YD3T56Z4Y2.1.

Post hoc power analysis was conducted to evaluate the statistical power of the study. Effect sizes (Cohen’s d) were calculated using IBM SPSS Statistics (version 29), based on group means and pooled standard deviations. These values were then used in G*Power (version 3.1.9.7, Heinrich-Heine-Universität Düsseldorf, Germany) to compute achieved power (1-β) for each region and modality using a 2-tailed t-test model at a significance level of α = 0.05. In addition to the post hoc power analysis, an a priori sample size calculation was conducted using G*Power software (version 3.1.9.7, Heinrich-Heine-Universität Düsseldorf, Germany). The calculation was based on a 2-tailed t-test for independent samples, with an alpha level of 0.05 and a desired power of 0.80 (1-β). Effect sizes (Cohen’s d) were estimated from the observed differences in FD and lacunarity values between the groups. The required sample size was determined separately for each region and imaging modality.

## Results

**Fig. 4:**
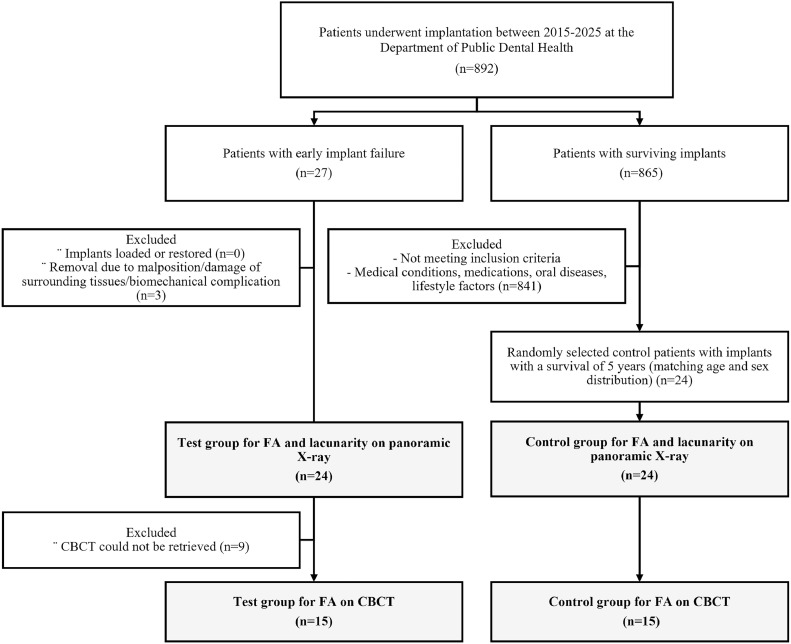
Flowchart of patient selection. Overview of the recruitment process for the retrospective case-control study, indicating the inclusion and exclusion criteria applied to form the test and control groups.

The patient selection criteria are shown in the flowchart below (Figure 4).

Descriptive data for the population are given in the first table (Table 1).

**Table 1:** Descriptive data of the test and control group.

Variable	Test group panoramic radiograph	Control Group panoramic radiograph	Test group CBCT	Control group CBCT
N	24	24	15	15
Age mean (year)	63.25 ± 17.38	60.67 ± 17.3	57.4 ± 18.13	63.13 ± 15.9
Gender				
Female	12	12	8	9
Male	12	12	7	6
Implant location (total number of implants placed)				
Maxilla front	17	4	14	2
Maxilla premolar	20	6	13	5
Maxilla molar	9	5	4	5
Mandible front	10	17	4	14
Mandible premolar	10	3	9	2
Mandible molar	11	9	9	3
Total	77	44	53	31
Implant location (total number of failed implants)				
Maxilla front	5	-	4	-
Maxilla premolar	7	-	3	-
Maxilla molar	6	-	4	-
Mandibula front	2	-	0	-
Mandibula premolar	2	-	3	-
Mandibula molar	7	-	4	-
Total	29	-	18	-

### Fractal dimension (FD) and lacunarity in 2D X-ray images

The median FD was 1.4687 (1.2576–1.6147) and 1.4567 (1.3350–1.6577) in the front region; the mean FD 1.4825 ± 0.0684 (95% CI = 1.4536-1.5114) and 1.4929 ± 0.0619 (95% CI = 1.4667–1.5190) in the premolar region; and the median FD 1.5218 (1.3373–1.6220) and 1.5085 (1.3190–1.5770) in the molar region for the test and control groups, respectively. The values of lacunarity were: the mean lacunarity was 0.1846 ± 0.0378 (95% CI = 0.1686–0.2006) and 0.1860 ± 0.0373 (95% CI = 0.1703–0.2018) in the frontal region; the median lacunarity was 0.1731 (0.1262–0.2432) and 0.1691 (0.1257–0.2503) in the premolar region; and the mean lacunarity was 0.1768 ± 0.0378 (95% CI = 0.1608–0.1927) and 0.1885 ± 0.0494 (95% CI = 0.1677–0.2094) in the molar region for the control and test group, respectively. No significant difference was found between the FD and lacunarity values of the test and control groups (*P > .*05) (Table 2).

**Table 2:** Panoramic X-ray fractal dimension and lacunarity values.

		Test group	Control group	P-value
N		24	24	
Front	FD median	1.4692	1.4472	.149†
	Range width for FD	0.3571	0.3227	
	Minimum	1.2576	1.3350	
	Maximum	1.6147	1.6577	
	Lacunarity mean	0.1846	0.1860	.895*
	Standard deviation for lacunarity	0.0378	0.0373	
Premolar	FD mean	1.4825	1.4929	.583*
	Standard deviation for FD	0.0684	0.0619	
	Lacunarity median	0.1731	0.1691	.951†
	Range width for lacunarity	0.1170	0.1246	
	Minimum	0.1080	0.1114	
	Maximum	0.2694	0.2879	
Molar	FD median	1.5218	1.5085	.143†
	Range width for FD	0.3734	0.3243	
	Minimum	1.2928	1.2764	
	Maximum	1.6662	1.6007	
	Lacunarity mean	0.1768	0.1885	.358*
	Standard deviation for lacunarity	0.0378	0.0494	

⁎ t-test.

† Mann-Whitney-U test

### Fractal dimension (FD) in 3D CBCTs

The FD values measured according to White and Rudoph^20^ were the following. The median FD was 1.8352 (1.3287–2.1461) and 1.9179 (1.3761–2.1628) in the front; 1.8452 (1.4732–2.2448) and 1.9006 (1.6388–2.9223) in the premolar region; and 1.6137 (1.2950–2.1065) and 1.8793 (0.9111–2.1170) in the molar region for the test and control group, respectively. No significant difference was found between the test and control groups in FD values (*P > .*05).

The median FD values measured by the method of Kato et al.^38^ were 1.8810 (1.6773–2.3028) and 2.0767 (1.4828–2.2667) in the front region; 1.9946 (0.9431–2.3442) and 1.9757 (1.6355–2.3064) in the premolar region and the mean FD was 1.8131 ± 0.3336 (95%CI = 1.6284–1.9978) and 1.8966 ± 0.3087 (95% CI = 1.7256–2.0675) in the molar region in the test and the control group, respectively. No significant difference was found between FD values measured in the test and control groups (*P* > .05) (Table 3).

**Table 3:** Results of fractal analysis for CBCTs.

		Test group	Control group	P-value
White and Rudoph's method				
		15	15	
Front	FD median	1.8352	1.9179	.917†
	Range width	0.8174	0.7867	
	Minimum	1.3287	1.3761	
	Maximum	2.1461	2.1628	
Premolar	FD median	1.8452	1.9006	.633†
	Range width	0.7716	1.2835	
	Minimum	1.4732	1.6388	
	Maximum	2.2448	2.9223	
Molar	FD median	1.6137	1.8793	.059†
	Range width	0.8115	1.2059	
	Minimum	1.2950	0.9111	
	Maximum	2.1065	2.1170	
Method of Kato et al.				
N		15	15	
Front	FD median	1.8810	2.0767	.330†
	Range	0.6255	0.7839	
	Minimum	1.6773	1.4828	
	Maximum	2.3028	2.2667	
Premolar	FD median	1.9946	1.9757	.724†
	Range	1.4011	0.6709	
	Minimum	0.9431	1.6355	
	Maximum	2.3442	2.3064	
Molar	FD mean	1.8131	1.8966	.483*
	Standard deviation	0.3336	0.3087	

⁎ t-test.

† Mann-Whitney-U.

Post hoc power analysis was conducted to assess the statistical power of the comparisons. The achieved power (1-β) values varied across regions and modalities, ranging from 5.1% to 42.2%, depending on the observed effect sizes and sample sizes. The a priori sample size calculation indicated that the number of patients required to achieve 80% power at α = 0.05 varied substantially depending on the effect size observed in each region. For instance, detecting a difference in FD in the molar region (Cohen’s d = 0.52) would require a total of 120 patients (60 per group). In contrast, regions with smaller effect sizes, such as the premolar FD (d =-−0.16), would require up to 1230 patients. For lacunarity and certain CBCT-based analyses, the required sample size exceeded 400-600 patients or more, reflecting the low sensitivity of these comparisons with the current sample. A summary of power values and sample size calculations for each region is provided in Supplementary table 2.

## Discussion

The present study aimed to investigate whether fractal dimension (FD) values—and, in panoramic radiographs, lacunarity values—could serve as indicators for early dental implant failure. The results of the present study supported our null hypothesis as no statistically significant differences were found in FD values between patients who experienced early implant loss and those with established early bone implant contact, in either 2D panoramic radiographs or 3D CBCT images. Similarly, no significant differences were observed in lacunarity values measured from panoramic radiographs.

Lee et al. found a significant correlation between FD and implant stability quotient scores in a clinical cohort.^24^ Furthermore, study such as that by Ünlü Kurşun & Akan has demonstrated that FD can sensitively detect trabecular changes in response to prosthetic loading and systemic conditions, emphasizing its potential role in monitoring post-implant bone dynamics.^25^ However, according to the study by Lang et al., the effects of peri-implant inflammation on bone cannot be simulated by FD.^26^ Several previous studies have demonstrated a relationship between FD values and implant stability or success, often focusing on the bone structure immediately adjacent to the implant site.^22^^,^^24^^,^^25^ In contrast, our investigation assessed the general trabecular bone structure of the mandible—analyzing anterior, premolar, and molar regions—rather than the direct peri-implant bone. This broader approach was selected to reflect systemic bone quality, which plays a central role in achieving primary implant stability and successful osseointegration.

Importantly, there is evidence in the literature that systemic bone conditions such as osteoporosis—a known risk factor for implant failure—are associated with quantifiable changes in bone trabecular patterns, detectable by fractal analysis.^15^^,^^20^^,^^39^, ^40^, ^41^ Studies have shown that patients with reduced bone mass or altered trabecular connectivity exhibit significantly lower FD values.^15^^,^^20^^,^^21^^,^^42^ These findings underscore the clinical relevance of generalized bone quality, not only in terms of biomechanical support but also as an indicator of osseointegration potential and resistance to early postoperative complications. Although the patient cohort enrolled in this study excluded individuals with diagnosed systemic bone diseases, our approach still aimed to capture this general architectural capacity of the mandibular bone, which may subtly influence implant prognosis even in the absence of clear pathological lesions or disease.

Therefore, we propose that analyzing the overall bone architecture—beyond the peri-implant region—provides valuable insight into a patient’s baseline bone condition. While local peri-implant bone morphology is undoubtedly critical for mechanical stability, it does not exist in isolation.^14^ Regional and systemic bone characteristics are inherently linked and may contribute synergistically to the biological processes that determine implant integration and long-term survival.^14^^,^^43^^,^^44^

The lack of statistically significant differences in our study may also be affected by technical aspects of fractal analysis. While we applied validated and widely accepted methods—including the protocol described by White and Rudolph,^20^ which remains a standard in the field—it is known that fractal dimension values can be sensitive to several factors such as ROI selection, image resolution, voxel size, and image preprocessing parameters.^45^^,^^46^ The sigma value used in Gaussian blur filtering significantly influences the degree of noise reduction applied to radiological images, as a higher sigma removes more low-frequency background while potentially eroding fine trabecular details.^47^ Since optimal sigma depends on the image resolution, ROI size, and noise level, differences in these parameters across studies may contribute to variability in fractal dimension outcomes. These factors do not invalidate the findings, but they may contribute to variability and should be carefully considered in study design and interpretation. This highlights the ongoing need for further methodological standardization, particularly in the context of 3-dimensional image analysis.^23^^,^^46^^,^^48^

From a clinical perspective, our findings suggest that FD and lacunarity, while offering important insights into bone microarchitecture, should not yet be interpreted as standalone diagnostic tools. Instead, these parameters may serve as useful adjuncts within a broader diagnostic context that includes radiographic density measurements, cortical thickness assessment, and systemic patient factors.

The present study is not without limitations. The retrospective design and relatively small sample size—especially in the CBCT subgroup—limit the generalizability of our findings. Given the retrospective nature of the study and the rarity of early implant failure, recruiting a large patient cohort is inherently difficult. Post hoc power analysis and sample size calculations showed that the statistical power to detect differences in FD and lacunarity was generally low across regions. These findings suggest that some non-significant results may be attributed to insufficient power rather than the absence of a true effect. Future studies with larger sample sizes are needed to validate these findings and detect more subtle differences in bone architecture. Additionally, while assessing general mandibular bone structure allowed us to evaluate systemic bone quality, it did not directly account for localized mechanical stresses or surgical variables at the implant site. Nonetheless, the approach used in this study contributes to the increasing understanding that implant survival is influenced not only by local conditions but also by the overall bone environment.

Future studies should aim to integrate both generalized and site-specific assessments of bone quality, possibly incorporating automated ROI selection and machine learning—based fractal analysis. Moreover, combining fractal parameters with other objective bone metrics—such as HU, ISQ or bone area fraction, could improve the predictive capacity for early implant outcomes.^49^ Although ISQ and HU values have shown promise as indicators of bone quality, these data were not consistently available in our retrospective sample. Furthermore, incorporating HU thresholds would have significantly reduced the CBCT subsample size. Future prospective studies may benefit from combining radiodensity and implant stability measures with fractal and lacunarity analysis to provide a more comprehensive evaluation.

Additionally, although CBCT offers 3-dimensional imaging capabilities, the majority of existing studies still perform fractal analysis on extracted 2-dimensional slices rather than on true volumetric datasets. Only a few investigations have applied genuine 3D fractal analysis techniques, and this methodological inconsistency further complicates cross-study comparisons. Our study applied a volumetric analysis approach in CBCT, aiming to capture trabecular bone architecture in a more comprehensive manner.^23^^,^^46^^,^^48^

## Conclusions

Within the limitation of this study, the results suggest that fractal and lacunarity analysis alone are not reliable predictors of early implant failure. The findings of this study highlight the potential of fractal and lacunarity analysis as valuable tools for assessing early implant outcomes. Although further methodological refinement is needed, fractal analysis already offers a promising approach for evaluating trabecular bone quality. With continued research and development, these techniques may become reliable and effective components in the preoperative assessment of implant candidates.

## Funding

This research did not receive any specific grant from funding agencies in the public, commercial, or not-for-profit sectors.

## Data availability

The datasets generated and analyzed during the current study are publicly available in the Mendeley Data repository: Iványi, Dóra; Kivovics, Márton; Balázs, Richárd; Németh, Orsolya (2025), “Data from: Is fractal analysis a reliable indicator for predicting early implant loss? A retrospective study.”, Mendeley Data, V1, doi: 10.17632/yd3t56z4y2.1

## Declaration of generative AI and AI-assisted technologies in the writing process

During the preparation of this work the authors used OpenAI's ChatGPT in order to assist with language refinement and grammar improvement during manuscript preparation. After using this tool, the authors reviewed and edited the content as needed and take full responsibility for the content of the publication.

## Author’s contributors

Dóra Iványi: Conceptualization, Methodology, Formal analysis, Investigation, Resources, Data Curation, Writing - Original Draft, Writing - Review & Editing. Márton Kivovics: Conceptualization, Validation, Formal analysis, Writing - Review & Editing. Richárd Balázs: Methodology, Investigation, Data Curation. Orsolya Németh: Conceptualization, Validation, Resources, Supervision, Project administration. All authors have approved the final version of the manuscript. All authors meet the International Dental Journal’s authorship criteria.

## Conflict of interest

None disclosed.

## Appendix Supplementary materials

Image, application 1

Image, application 2

## Footnotes

Supplementary material associated with this article can be found in the online version at doi:10.1016/j.identj.2025.103880.
