# Supplementary material for: Fractal Analysis as a Predictor of Early Implant Loss: A Retrospective Study
Source: Int Dent J. 2025 Aug 30;75(6):103880. doi: 10.1016/j.identj.2025.103880 (PMC12414887; doi:10.1016/j.identj.2025.103880)
Supplement: Supplementary file 1 [file mmc1.docx]

STROBE Statement—checklist of items that should be included in reports of observational studies

|  | Item No. | Recommendation | Page  No. | Relevant text from manuscript |
| --- | --- | --- | --- | --- |
| **Title and abstract** | 1 | (*a*) Indicate the study’s design with a commonly used term in the title or the abstract | Title | The title clearly indicates a retrospective case-control study design. The abstract provides a balanced summary of objectives, methods, key results, and conclusions. |
|  |  | (*b*) Provide in the abstract an informative and balanced summary of what was done and what was found | Abstract | Background: Fractal dimension (FD) and lacunarity are increasingly applied to assess trabecular bone structure, yet their predictive value for early dental implant failure remains uncertain. Objective: This study investigated whether FD and lacunarity, measured on panoramic radiographs and cone-beam computed tomography (CBCT), can predict early implant loss in the mandible. Materials and Methods: A retrospective case-control study included 48 patients—24 with early implant failure and 24 with implants surviving at least 5 years without periimplantitis. All had panoramic radiographs, and a subset of 30 patients (15 per group) also had CBCT scans. FD and lacunarity were assessed in three mandibular regions: frontal, premolar, and molar. ImageJ software with box-counting method was used. FD from panoramic images was calculated using the White and Rudolph method; CBCT-based FD values were derived using both the White and Rudolph and the Kato et al. methods. Results: No significant differences in FD or lacunarity values were found between groups in any region on either imaging modality (p > 0.05). Conclusions: Fractal dimension and lacunarity values obtained from panoramic radiographs and CBCT images did not demonstrate predictive capability for early implant failure. Although fractal analysis offers a non-invasive approach to assess trabecular bone architecture, it should not be used in isolation to estimate implant prognosis. Clinical Significance: While fractal analysis may provide complementary insights into bone microstructure, its standalone application lacks sufficient reliability for predicting early dental implant failure. Future studies involving larger cohorts are warranted. |
| Introduction | | | |  |
| Background/rationale | 2 | Explain the scientific background and rationale for the investigation being reported | Introduction | The introduction explains the clinical relevance of early implant failure and the potential role of fractal analysis in evaluating bone quality. |
| Objectives | 3 | State specific objectives, including any prespecified hypotheses | Introduction | The specific aim is to investigate whether FD and lacunarity measured on panoramic radiographs and CBCT can predict early implant failure. The null hypothesis is clearly stated. |
| Methods | | | |  |
| Study design | 4 | Present key elements of study design early in the paper | Materials and methods, Abstract | A retrospective case-control design was used and mentioned in the abstract and introduction. |
| Setting | 5 | Describe the setting, locations, and relevant dates, including periods of recruitment, exposure, follow-up, and data collection | Materials and methods, Participants | The study was conducted at Semmelweis University between 2015 and 2025. Data were collected and analysed in April 2025. |
| Participants | 6 | (*a*) *Cohort study*—Give the eligibility criteria, and the sources and methods of selection of participants. Describe methods of follow-up  *Case-control study*—Give the eligibility criteria, and the sources and methods of case ascertainment and control selection. Give the rationale for the choice of cases and controls  *Cross-sectional study*—Give the eligibility criteria, and the sources and methods of selection of participants | Materials and methods, Participants | Clear inclusion and exclusion criteria are listed for both case and control groups. Cases were patients with early implant failure; controls were patients with surviving implants for at least 5 years. |
|  |  | (*b*) *Cohort study*—For matched studies, give matching criteria and number of exposed and unexposed  *Case-control study*—For matched studies, give matching criteria and the number of controls per case |  | Not applicable – no individual matching was performed. |
| Variables | 7 | Clearly define all outcomes, exposures, predictors, potential confounders, and effect modifiers. Give diagnostic criteria, if applicable | Materials and methods | The main outcomes were FD and lacunarity values measured in three mandibular regions. Independent variables include group assignment (case/control) and imaging modality (panoramic vs. CBCT). |
| Data sources/ measurement | 8* | For each variable of interest, give sources of data and details of methods of assessment (measurement). Describe comparability of assessment methods if there is more than one group | Material*s* and methods, Radiographic image analysis | Imaging data were obtained from standardized panoramic radiographs and CBCT scans. Fractal analysis was performed using ImageJ software following validated protocols. |
| Bias | 9 | Describe any efforts to address potential sources of bias | Matherials and methods | Efforts to minimize bias included strict inclusion/exclusion criteria and standardized image processing protocols. |
| Study size | 10 | Explain how the study size was arrived at | Results | Sample size was determined by available cases meeting criteria within the study period. Equal group sizes (n=24 and n=15) were used. |

Continued on next page

| Quantitative variables | 11 | Explain how quantitative variables were handled in the analyses. If applicable, describe which groupings were chosen and why | Material and methods, Statistical analysis | Quantitative variables such as FD and lacunarity were treated as continuous variables. Normality of distribution was assessed using the Kolmogorov-Smirnov and Shapiro–Wilk tests. Based on distribution, appropriate statistical tests (e.g., independent samples t-test or Mann–Whitney U test) were used to compare values between groups. No categorical groupings were created from continuous variables. |
| --- | --- | --- | --- | --- |
| Statistical methods | 12 | (*a*) Describe all statistical methods, including those used to control for confounding | Material and methods, Statistical analysis | Normality was tested using Kolmogorov-Smirnov and Shapiro-Wilk tests. Depending on distribution, independent samples t-tests or Mann-Whitney U tests were used. A p-value < 0.05 was considered significant. |
|  |  | (*b*) Describe any methods used to examine subgroups and interactions |  | Not applicable – no subgroup or interaction analyses were performed. |
|  |  | (*c*) Explain how missing data were addressed |  | Participants with missing imaging or clinical data were excluded during initial screening as part of the study’s exclusion criteria. Therefore, missing data did not affect the final analyses. |
|  |  | (*d*) *Cohort study*—If applicable, explain how loss to follow-up was addressed  *Case-control study*—If applicable, explain how matching of cases and controls was addressed  *Cross-sectional study*—If applicable, describe analytical methods taking account of sampling strategy |  | A retrospective case-control study was conducted to compare patients with early implant failure to those with successful long-term outcomes. Groups were selected to have broadly similar age and sex distributions. |
|  |  | (*e*) Describe any sensitivity analyses |  | Not applicable – no sensitivity analyses were conducted. |
| Results | | | | |
| Participants | 13* | (a) Report numbers of individuals at each stage of study—eg numbers potentially eligible, examined for eligibility, confirmed eligible, included in the study, completing follow-up, and analysed | Results | A flowchart was included to show patient selection. |
|  |  | (b) Give reasons for non-participation at each stage | Results | Reasons for exclusion were based on missing imaging data or exclusion criteria. |
|  |  | (c) Consider use of a flow diagram | Results | Figure 4 |
| Descriptive data | 14* | (a) Give characteristics of study participants (eg demographic, clinical, social) and information on exposures and potential confounders | Results | Demographic and clinical characteristics are presented in Table 1, including age, gender, and implant distribution. |
|  |  | (b) Indicate number of participants with missing data for each variable of interest |  | Participants with missing imaging or clinical data were excluded during initial screening as part of the study’s exclusion criteria. Therefore, missing data did not affect the final analyses. |
|  |  | (c) *Cohort study*—Summarise follow-up time (eg, average and total amount) |  |  |
| Outcome data | 15* | *Cohort study*—Report numbers of outcome events or summary measures over time |  |  |
|  |  | *Case-control study—*Report numbers in each exposure category, or summary measures of exposure | *Results* | Results for FD and lacunarity are presented in Tables 2 and 3 for each anatomical region and imaging modality. |
|  |  | *Cross-sectional study—*Report numbers of outcome events or summary measures |  |  |
| Main results | 16 | (*a*) Give unadjusted estimates and, if applicable, confounder-adjusted estimates and their precision (eg, 95% confidence interval). Make clear which confounders were adjusted for and why they were included | Restults | No statistically significant differences were found in FD or lacunarity between groups. Results are reported with p-values and descriptive statistics.  Unadjusted comparisons between groups were performed using t-tests or Mann–Whitney U tests, depending on data distribution. 95% confidence intervals were calculated and reported for variables analyzed with parametric tests. No confidence intervals were provided for non-parametric tests, in line with standard reporting practice. |
|  |  | (*b*) Report category boundaries when continuous variables were categorized |  | No continuous variables were categorized for analysis; all were used as continuous measures. |
|  |  | (*c*) If relevant, consider translating estimates of relative risk into absolute risk for a meaningful time period |  | Not applicable. No relative or absolute risk estimates were calculated in this study. |

Continued on next page

| Other analyses | 17 | Report other analyses done—eg analyses of subgroups and interactions, and sensitivity analyses |  | No subgroup or adjusted analyses were conducted due to the limited sample size. |
| --- | --- | --- | --- | --- |
| Discussion | | | | |
| Key results | 18 | Summarise key results with reference to study objectives | Discussion | The study found no predictive value for FD and lacunarity in early implant failure. The null hypothesis was confirmed. |
| Limitations | 19 | Discuss limitations of the study, taking into account sources of potential bias or imprecision. Discuss both direction and magnitude of any potential bias | Discussion | Limitations include the small sample size, retrospective design, and limited generalizability due to single-centre data. |
| Interpretation | 20 | Give a cautious overall interpretation of results considering objectives, limitations, multiplicity of analyses, results from similar studies, and other relevant evidence | Discussion | The results are cautiously interpreted in the context of existing literature. Potential sources of variability and the need for standardization are discussed. |
| Generalisability | 21 | Discuss the generalisability (external validity) of the study results | Discussion | The generalisability of the findings is limited by the study’s retrospective design, the relatively small sample size, and the inclusion of patients from a single academic center. These factors may restrict the applicability of the results to broader populations or different clinical settings.  Nevertheless, the use of standardized image acquisition and analysis protocols, and the inclusion of both panoramic and CBCT imaging modalities, support the potential reproducibility of the findings in similar clinical environments. |
| Other information | |  | | |
| Funding | 22 | Give the source of funding and the role of the funders for the present study and, if applicable, for the original study on which the present article is based | Funding | No funding source was reported. |

*Give information separately for cases and controls in case-control studies and, if applicable, for exposed and unexposed groups in cohort and cross-sectional studies.

**Note:** An Explanation and Elaboration article discusses each checklist item and gives methodological background and published examples of transparent reporting. The STROBE checklist is best used in conjunction with this article (freely available on the Web sites of PLoS Medicine at http://www.plosmedicine.org/, Annals of Internal Medicine at http://www.annals.org/, and Epidemiology at http://www.epidem.com/). Information on the STROBE Initiative is available at www.strobe-statement.org.
