# Supplementary material for: Fractal Analysis as a Predictor of Early Implant Loss: A Retrospective Study
Source: Int Dent J. 2025 Aug 30;75(6):103880. doi: 10.1016/j.identj.2025.103880 (PMC12414887; doi:10.1016/j.identj.2025.103880)
Supplement: Supplementary file 2 [file mmc2.docx]

| **Region / Method** | **Cohen’s d** | **Power (1–β)** | **Sample size*** |
| --- | --- | --- | --- |
| **Panoramic – FD** |  |  |  |
| Front | 0.34 | 21.1% | 274 (137 per group) |
| Premolar | -0.16 | 8.4% | 1230 (615 per group) |
| Molar | 0.52 | 42.2% | 120 (60 per group) |
| **Panoramic – Lacunarity** |  |  |  |
| Front | -0.04 | 5.2% | 19626 (9813 per group) |
| Premolar | -0.08 | 5.8% | 4908 (2454 per group) |
| Molar | -0.27 | 15.0% | 434 (217 per group) |
| **CBCT Method I – FD (White&Rudolph)** |  |  |  |
| Front | 0.04 | 5.1% | 19626 (9813 per group) |
| Premolar | -0.33 | 14.1% | 292 (146 per group) |
| Molar | -0.52 | 28.0% | 120 (60 per group) |
| **CBCT Method II – FD (Kato et. al)** |  |  |  |
| Front | -0.28 | 11.5% | 404 (202 per group) |
| Premolar | -0.07 | 5.4% | 6410 (3205 per group) |
| Molar | -0.26 | 10.6% | 468 (234 per group) |
| ***Power (1-β err prob)=80%** | | | |

*Supplementary Table 2. Results of power analysis.*
